# Sustaining Work Participation Across the Life Course

**DOI:** 10.1007/s10926-016-9670-1

**Published:** 2016-10-04

**Authors:** Glenn S. Pransky, Jean-Baptise Fassier, Elyssa Besen, Peter Blanck, Kerstin Ekberg, Michael Feuerstein, Fehmidah Munir, the Hopkinton Conference Working Group on Workplace Disability Prevention, Benjamin C. Amick, Johannes R. Anema, Elyssa Besen, Peter Blanck, Cécile R. L. Boot, Ute Bültmann, Chetwyn C. H. Chan, George L. Delclos, Kerstin Ekberg, Mark G. Ehrhart, Jean-Baptiste Fassier, Michael Feuerstein, David Gimeno, Vicki L. Kristman, Steven J. Linton, Chris J. Main, Fehmidah Munir, Michael K. Nicholas, Glenn Pransky, William S. Shaw, Michael J. Sullivan, Lois E. Tetrick, Torill H. Tveito, Eira Viikari-Juntura, Kelly Williams-Whitt, Amanda E. Young

**Affiliations:** 1 Liberty Mutual Research Institute for Safety, 71 Frankland Road, Hopkinton, MA 01748 USA; 2 University of Massachusetts Medical School, Worcester, MA USA; 3 Burton Blatt Institute, Syracuse University, Syracuse, NY USA; 4 Linköping University, Linköping, Sweden; 5 Claude Bernard University Lyon 1, Lyon, France; 6 Uniformed Services University of the Health Sciences, Bethesda, MD USA; 7 University of Loughborough, Leicester, UK

**Keywords:** Chronic health conditions, Employer practices, Cancer, Mental health

## Abstract

*Introduction* Many disability prevention strategies are focused on acute injuries and brief illness episodes, but there will be growing challenges for employers to manage circumstances of recurrent, chronic, or fluctuating symptoms in an aging workforce. The goal of this article is to summarize existing peer-review research in this area, compare this with employer discourse in the grey literature, and recommend future research priorities. *Methods* The authors participated in a year-long sponsored collaboration that ultimately led to an invited 3-day conference, “Improving Research of Employer Practices to Prevent Disability”, held October 14–16, 2015, in Hopkinton, Massachusetts, USA. The collaboration included a topical review of the scientific and industry literature, group discussion to identify key areas and challenges, drafting of initial documents, and feedback from peer researchers and a special panel of experts with employer experience. *Results* Cancer and mental illness were chosen as examples of chronic or recurring conditions that might challenge conventional workplace return-to-work practices. Workplace problems identified in the literature included fatigue, emotional exhaustion, poor supervisor and co-worker support, stigma, discrimination, and difficulties finding appropriate accommodations. Workplace intervention research is generally lacking, but there is preliminary support for improving workplace self-management strategies, collaborative problem-solving, and providing checklists and other tools for job accommodation, ideas echoed in the literature directed toward employers. Research might be improved by following workers from an earlier stage of developing workplace concerns. *Conclusions* Future research of work disability should focus on earlier identification of at-risk workers with chronic conditions, the use of more innovative and flexible accommodation strategies matched to specific functional losses, stronger integration of the workplace into on-going rehabilitation efforts, and a better understanding of stigma and other social factors at work.

## Introduction

One profound demographic shift facing many industrialized nations is the increasing longevity and average age of the population. As the population ages, the available workforce is also aging. By 2020, at least quarter of the workforce in many countries is expected to be age 55 and older [1]. At the same time, chronic health conditions are becoming increasingly important as a cause of work disability across the globe, affecting not only aging workers, but also youth in transition, and mid-career workers. Over 40 % of US workers have a chronic health condition and 15–20 % of workers report health-related work limitations; studies in European countries yield similar results [2]. These shifts in the age and fitness of workers are likely to influence employer practices to manage and prevent disability.

Research of employer practices and their impact on health and disability may be improved by adopting a life-course perspective on employee health and disability. A life-course view examines how chronological age, changing relationships, life transitions, social and occupation development shape people’s lives from birth to death [3]. This perspective creates an opportunity to examine how the impact of workplace factors and workplace responses to work disability might differ depending on the age and career stage of a worker, and where particular workplace WDP approaches may be more important.

Although some work disability problems observed in aging workers are primarily related to health, other factors—such as competing retirement options, career status, likelihood of accommodations and mobility in the workforce—are specific to age group and career stage, and can interact with health [3]. Economic, social and demographic changes have led to an increased need to include more older workers in the workforce, and to find ways to better enable workers with health conditions to maintain employment [4]. This direction is supported by new legislation that seeks to support the rights and ability to work of persons with potentially disabling conditions, as exemplified by the UN Convention of the Rights of Persons with Disabilities (CRPD), stating that workers with disabilities have an equal opportunity to employment [5]. The rights of disabled people set out in the CRPD presently are recognized by more than one hundred and fifty nations who ratified the treaty [5]. These developments all suggest the need for more information on how workplace factors and related interventions can better support the employment of persons with various health conditions, across the life-course.

There are fundamental principles and effective strategies for work disability prevention (WDP) that appear to be common across health conditions and work situations, and are consistent with the goals of the CRPD. Some of these principles and strategies include providing appropriate health care, establishing workers’ ability to do meaningful work, promoting employer responsiveness, and offering job accommodations [6]. However, most research on workplace issues and WDP has focused on musculoskeletal disorders and work injuries, there is less evidence for generalization of these principles across other types of health conditions [7]. Our objective was to identify relevant scientific research and current employer practices for managing chronic health conditions and related work disability in the workplace, as a basis for recommendations for improving future research of employer disability prevention strategies.

## Methods

The authors participated in an invited 3-day conference, “Improving Research of Employer Practices to Prevent Disability”, held October 14–16, 2015, in Hopkinton, Massachusetts, USA. Methods and general proceedings of the conference are described in the introductory article to this special issue [8]. The authors of this article represented a sub-group tasked with understanding the state of the science with respect to employer practices for managing the increasing prevalence of chronic health conditions and workplace efforts to prevent this source of disability through job accommodation and support, through a life-course view of employment and disability. We contrast key conceptual and theoretical frameworks, review the applicable scientific literature, assess its impact for employer decision-making, and compare recommendations with that of the employer-directed grey literature. These observations and recommendations are useful for areas that have not been thoroughly investigated. We recommend future research priorities, based on important scientific gaps. Two chronic health conditions with divergent prevalence across the life span—mental disorders [9] and cancer [10]—were chosen as exemplars to illustrate key principles that may apply across a range of conditions.

## Results

### Aging and Chronic Illness in the Workplace

As a result of decreasing fertility and mortality rates, coupled with increasing life expectancy, the percentage of the world’s population age 60 years and older is expected to increase from 9.2 % in 1990 to 21.1 % by 2050 [11]. In OECD countries, the labor force participation rate of workers ages 55–64 years has increased from 47.7 % in 1990 to 55.6 % by 2012 [12]. Although there are significant differences by country, retirement ages are gradually increasing in most established economies. In older workers, chronological age may be inconsistent with career stage and status. Some older workers may be nearing retirement, and others are already in post-retirement careers, and thus may have very different work disability considerations. Post-retirement workers may have lower job attachment, less financial or personal need to return to work after a disabling illness, and may be more interested in exploring alternative employment—especially if the job is perceived as a poor match for their capabilities. Older workers may want to stay at work for the benefits of social engagement and discretionary income. In many countries, employers need these workers, and are especially interested in capturing their expertise and maturity [13]. However, the growth of an aging workforce has heightened employer concerns about the ability of older workers to remain at work given their higher rate of medical illness, and increased risk for work disability [10, 14].

#### Impact of Chronic Health Conditions in the Workplace

Chronic health conditions—including obesity, diabetes, mental disorders, cancer, arthritis, and lung, gastrointestinal and cardiovascular diseases—account for considerable work disability (WD), and reduced productivity on the job [15, 16]. Their prevalence in the workforce is changing for several reasons. Advances in medical care is converting mortality to morbidity. Workers with chronic illness have fewer resources enabling them to retire, and the impacts of a sedentary lifestyle are becoming more manifest in older workers [17]. Many workers with chronic illness have multiple comorbidities or simultaneous conditions, challenging traditional views of a single disease as a cause of work disability, and are therefore at higher risk of frequent or prolonged work absence [18].

Several studies have examined the specific challenges that workers with chronic health conditions face at work, that place them at risk for low productivity or WD. These problems include fatigue, emotional exhaustion, inconsistent support from colleagues and supervisors, and difficulty modifying their jobs to match their capabilities [19]. With serious chronic illness, medication adherence and side-effects can be additional risk factors for lower productivity and work absence [20]. Although stigma has been primarily associated with mental illness, the problem of stigma and discrimination at work is reported across a range of chronic conditions, and interventions to educate employers and co-workers are a major emphasis of condition-specific advocacy groups for diabetes, lung disease, cancer and other conditions.

Research has shown that while older workers are not at an increased risk for a work-disabling injury [10, 21], when one does occur, older workers frequently sustain more serious injuries than younger workers, they take longer to RTW, are more likely to leave work again after attempting to RTW, and have a higher chance of never achieving a successful RTW [14, 22] Work disability claims for older workers tend to be more costly than for younger workers [23], adding further concerns for employers.

#### Interventions to Promote Retention and Productivity

The traditional workplace approach to address work disability and chronic illness focuses on health improvement, disease management, in systems directed by legal requirements, administrative structures, diagnostic classifications, and specific rules [23]. Despite the inverse relationship between age and positive RTW outcomes, there have been relatively few workplace interventions to improve RTW targeting older workers (pre or post retirement) on those with chronic illness of any age. It is well known that employer attitudes, organizational culture and support play a critical role in older workers’ RTW [24], but these factors have not been evaluated in comparative trials. For example, employer responses to work-related and non-work related conditions are often different, with respect to inquiry about causation, concerns about liability, case management practices, and nature of accommodations to facilitate return to work [25]. Condition-specific employment advocacy groups promote strategies to support employment of younger workers with chronic health conditions, but these lack empirical evidence of where and when they are helpful or effective.

#### Fitness and Wellness Programs

Although frequently advocated in the professional literature, general workplace fitness and lifestyle programs have limited impact on WD rates [23, 26], even those targeting physical activity and fitness as a way of decreasing sickness-related absence [27]. These wellness programs usually fail to engage those who could most benefit, and few have documented long-term sustained health benefits in large numbers of enrollees. For example, one workplace intervention involved exercise, coaching related to vitality, and healthy eating, targeting workers over the age of 65 [28], and was not found to be cost-effective. In contrast, an intervention involving food industry workers aged 55 and older aimed at maintaining well-being and work status found that absenteeism of 21 days or longer was decreased [29]. Another study involving female nurses age 49 or older, found that a Tai Chi workplace intervention reduced perceived work limitations compared to no intervention [30].

#### Medical Case Management

Diagnosis—based medical case management that focuses primarily on improving clinical care and compliance, without substantial integration of workplace issues, is generally not effective in preventing work disability or improving RTW [31, 32]. This approach fails to take into account the wide variation in work ability within a single diagnosis, and nonmedical (including workplace) factors that primarily determine RTW outcomes. There are a few exceptions—for example, screening and initiating treatment for an untreated, severe, and potentially-disabling condition (e.g., major depression or untreated diabetes) that has already resulted in significant functional impairment does improve WD outcomes [33].

#### Identifying Workers at Greatest Risk of Disability

There are a few examples of individualized, workplace-based programs that do effectively target WD risk. For example, Kant, Jansen et al [34] describe an intervention at a large Dutch bank, where risk for WD was identified through a screening tool, and high-risk persons were referred to an occupational physician for consultation, development of a personalized program to address workplace and individual risk factors, and follow-up. Compared to controls, the intervention group had 13 (about 30 %) fewer lost work days. About half of the participants had a chronic illness (cardiovascular, musculoskeletal, and other conditions), although results were not reported for this group separately. An intervention that addressed lifestyle and work in a comprehensive, individualized manner, involving the workplace, for persons with diabetes and obesity, had a significant impact on lost work days in a randomized trial [35]. A similar trial, with occupational physician consultation for workers at high risk for future (sickness absence), was reported in 2008 by Taimela et al [36]; although this study also included a significant number of workers with chronic health conditions (besides mental health problems), the exact numbers or effectiveness of the intervention with this subset was not reported. Together, these studies reinforce the value of an individualized workplace approach to sickness absence, taking into account the person, condition, and workplace, and addressing these aspects in an integrated manner. However, these studies have a clinical focus, and don’t change the workplace in a more fundamental way. There are no comparable scientific workplace studies of workers focusing on an individualized approach at either end of the age spectrum.

#### Strategies to Minimize Impact of Chronic Illness on Work

Studies of workers with chronic illness who are successful on the job, despite serious health problems suggest new strategies that may be effective. Workers with chronic pain reported that modifying work activities and routines, reducing pain symptoms, using cognitive strategies and communicating effectively about pain were important. Six predominant themes emerged: knowing your work setting, talking about pain, being prepared for a bad day, thoughts and emotions, keeping moving and finding leeway [37]. In another study, success factors were categorized into five themes: personal characteristics, adjustment latitude, coping with pain, use of healthcare services, and pain beliefs [38]. Studies of workers with chronic medical conditions identify supervisor support and ability to modify work as key factors [39]. In one study, employer representatives identified workplace factors that were most important in supporting continued employment for workers with chronic illness. Line managers focused on employer/employee cooperation as most important, whereas human resource managers focused more on the importance of organizational policy and culture related to working with a health condition [40].

Recently, interventions based on these observations have attempted to enhance worker self-management and supervisor support in the workplace. Results so far have indicated improved self-efficacy for staying at work, but no significant impact on RTW or work retention [41]. Another approach has focused on improving support in the workplace, through trained supervisors or a non-medical peer advocate, who provide reassurance, facilitate accommodations, and problem-solving. This strategy has been successful in decreasing work absence due to musculoskeletal pain, minor mental health conditions (anxiety) and minor gastrointestinal problems [42]. As accommodations are an essential component of work disability prevention in workers with chronic illness [43], workplaces are experimenting with a bottom-up non-medical strategy for workplace accommodations. In these models, employees and their supervisors have the primary responsibility for figuring out accommodations; grey literature reports cite greatly improved work absence outcomes [44]. Workplace DM policies and programs also struggle with intermittent work absence that can occur because of chronic illnesses, and generally focus on the administrative complexities of managing entitlements such as sick leave benefits, rather than the opportunity for coordinating medical care and workplace accommodation. As the functional impacts of chronic illness can vary greatly over time, an employee-driven approach may offer a more flexible and responsive solution than requesting a formal accommodation each time there is a change in health and functional ability, as long as the accommodations are reasonable for both employer and employee.

There has also been some effort to examine whether workplace interventions may be more effective at different ages. One study of individuals participating in a workplace intervention aimed at increasing RTW after taking sick-leave as a result of burnout showed that the intervention was only effective for younger workers (under 45.5 years of age) [45]. Another intervention aimed at improving RTW following low back pain was only effective for older workers [46]. Other studies examined potential age differences in the effectiveness of interventions but did not find differences based on participants’ ages [26].

### Cancer and Work

Cancer is one of the leading causes of morbidity and mortality worldwide. It is estimated that about 40 % of all cancer patients are diagnosed between ages 15 and 64 years, when work life plays a significant role [47]. Surviving cancer can lead to new challenges with regard to work. Many employees diagnosed and treated for cancer are interested in returning or remaining at work. This section provides a summary of cancer survivors and work in order to illustrate the current evidence related to this health problem at work. This section also addresses some of the age related concerns because as with most chronic health problems, cancer and its treatment can impact many different age groups of future and current employees.

#### Cancer and Return to Work

As with other serious health problems the first of these challenges is returning to work during or following cancer treatment. The evidence across studies suggests that an average of 63.5 % of cancer survivors do return to work [48].). However, return-to-work rates among cancer survivors vary from 24 to 94 %, depending on the type and stage of cancer and the treatment regime [48, 49]; comorbidity [50]; and perceptions of work ability [51]. The time taken to return to work also varies significantly with around 40 % returning to work by 6 months post diagnosis to around 89 % returning by 24 months post diagnosis [48]. The rate of recovery of cognitive work ability is gradual, highly variable, and can take years in some cases, although full recovery is often possible [52].

While return to work also varies by type of sick leave or national policy [53], unemployment remains a significant risk factor among cancer survivors [54]. Though the specific reasons for leaving the workplace were not clearly identified, a recent a review by Mehnert [48] found that between 26 and 53 % of cancer survivors either lost their job or left their job over a 72-month period post diagnosis although between 23 and 75 % of those were re-employed. Those recovering from breast and lung cancer are at higher risk of unemployment or reduced labour force participation [55]. These figures suggest that while the majority of cancer survivors do eventually return to work, remaining at work may pose a greater challenge [56].

#### Work Retention After Cancer

A number of long-term and late effects of cancer or its treatment have been related to poor work outcomes including work retention among cancer survivors. These include fatigue, physical and cognitive problems [57], and decreased mental and physical work ability [58]. Accordingly, there has been a focus across a number of countries on raising awareness among employers on the impact of cancer and its treatment upon employees [59, 60], and several conceptual models of cancer and work have been developed (Table 1).

**Table 1:** Conceptual models of cancer and work outcomes from the scientific literature

Author	Direct factors	Indirect factors	Mediating factors	Moderating factors	Outcome measures
Chow [91]	Financial pressure; health insurance; socio-demographic factors	Health status; personal factors and beliefs; environmental and workplace factors	Physical and mental work demands; physical and mental work ability	Physical and mental work demands interact with physical and mental work ability	Return to work
Feuerstein [92]	Work environment; work demands; policies and procedures; economic factors	Health and wellbeing, symptoms	Work environment; policies and procedures; economic factors	Health and wellbeing interacts with symptoms; functional status interacts with work demands	Return to work, work ability, work performance sustainability (work retention)
Mehnert [48]	Disease specific factors; treatment related factors	Disease specific factors; treatment related factors	Demographic factors; impairments; health status; psychosocial and motivational factors; work-related factors and interventions	Not described	Employment status; return-to-work; work ability; sick leave and duration; work changes; productivity; disability
Mehnert [48]	Interventions and rehabilitation programs promoting return to work and employment	Individual and interpersonal factors; short-term, long-term, and late effects of cancer and cancer treatments	Interventions and rehabilitation programs promoting return to work and employment	Individual and interpersonal factors; short-term, long-term, and late effects of cancer and cancer treatments	Employment/return to work; work ability; work performance; job opportunities; income; work satisfaction; job promotion and training; sustainability
Steiner [93]	Individual characteristics; general health perceptions; characteristics of work environment	Cancer and health status; symptoms; functional status	Health status; symptoms; functional status	Not described	Work status; job changes; work and role content; economic status; job satisfaction

Cancer survivors are more likely to be employed if they perceived their employer as accommodating, received support from their colleagues and line manager and had a good relationship with their employer/line manager [53, 61]. Another contributing factor to good work outcomes are actual work adjustments or accommodations. A study by Torp et al [58] found that slightly over a quarter of employed cancer survivors had work adjustments made such as reducing/changing the number of hours worked per week and changes to work tasks which enabled them to continue working.

Poorer work retention (i.e., cancer survivors changing jobs) among cancer survivors has been related to job discrimination [62]. In the US, when the workplace is perceived as creating an atmosphere where forms of discrimination related to work ability are alleged, an employee has a right to file a claim for employment discrimination (under the Americans with Disabilities Act). A study by Feuerstein et al [60] found that cancer survivors are more likely to file claims related to job loss or differential treatment by workplace policies than other chronic illness groups. Lack of understanding or support from supervisors affecting the work ability of cancer survivors has also been reported [63]. A UK study found wide diversity among organizations in their capacity to offer flexible arrangements and some employers failed to make adequate provisions for cancer survivors due to their lack of awareness regarding the needs of employees diagnosed with cancer [51]. Other factors associated with poor work ability and actual work retention were high job demands, and lack of promotion or career progression due to cancer [53, 58].

#### Workplace Based Interventions

To date, there is a paucity of workplace-based interventions specifically for those diagnosed and treated with cancer [64]. Munir et al [65] developed a behavior checklist to help supervisors return cancer survivors back to work. However, while anecdotal data suggest it is helpful and download data suggests people are interested in looking at the checklist, it has not been empirically tested for effectiveness. Munir et al [66] also developed a work self-management tool to be used by cancer survivors with their employers to identify needed workplace adjustments and support. While promising, these new tools need to be studied to determine their effectiveness. Two research protocols currently in progress [67, 68] focus on return to work in cancer and involve the workplace.

### Mental Health Conditions and Work

Most people with mental ill-health are affected by mild-to-moderate illness—predominantly mood and anxiety disorders, commonly referred to as “common mental illness”. According to the Global Burden of Diseases Study, mental disorders and substance abuse were the chief causes of years lived with disability (175 million years worldwide in 2010) [69]. In the European Union, the estimated total costs of mental illness were around 3.5 % of GDP in 2010, with similar estimates for developed non-European countries [70]. Mental ill-health affects one-fifth of the working-age population at any given moment [70]. In the US, a survey found a total of 6.4 % of employed respondents had a major depressive disorder episode in the past 12 months, and an additional 1.1 % had major depressive episodes due to bipolar disorder or mania-hypomania [71].

In OECD countries, mental ill-health is responsible for between one-third and one-half of all long-term sickness and disability among the working-age population [70]. In the US, 36 % of Social Security Disability Insurance (SSDI) and 60 % of working-age Supplemental Security Income (SSI) beneficiaries have a mental illness as their primary reason for inability to work [72]. Relative to the mentally healthy, the employment rate of people who suffer from poor mental health is 15–30 % lower and their unemployment compensation rate is twice as high [73]. Co-morbidity of back pain and common mental disorders is associated with a higher risk of disability pension than either individual condition, when added up [74].

In the workplace, mental ill-health has a greater financial impact due to low productivity at work than work absence. Approximately 30 % of lost work productivity due to depression in the US workforce is attributable to absenteeism, but 70 % is attributable to presenteeism [71]. For major depressive disorders (MDD), annualized estimates are 225 million lost workdays and $36.6 billion lost productivity per year in the US [71].

#### Mental Health and the Work Environment

Although work is a generally protective environment and fosters good mental health [75], it can also cause distress for certain employees and exacerbate mental health conditions [73]. Poor-quality jobs, problematic leadership, and psychosocial stress in the workplace can put the psychological health of the worker under strain and even trigger specific mental health conditions [76]. In the EU, high psychological demands, discrimination, bullying, and work-life imbalance have been observed as risk factors for sickness absence onset [77].

As with other chronic conditions, cause-and-effect relationships between the work environment and mental health are complex and multi-directional. On the one hand, poor psychosocial work environment can cause mental ill-health. On the other hand, workers with mental health problems tend to work in lower-quality jobs and environments, earn less per hour, have less secure jobs, are less satisfied with their jobs, report strain more often, and perceive their work situation more negatively [73]. The importance of psychosocial hazards in the work environment has been acknowledged by many countries through labor legislation requiring employers to routinely assess, prevent and control psychosocial risks at work. However, employers may consider the incentives for reducing psychosocial workplace risks as less compelling than for general workplace risks. Many enterprises, especially most small enterprises, receive minimal support or incentives to address psychosocial risks.

#### Interventions to Promote Return to Work and Work Retention

A wide range of interventions with a focus at the individual (worker), organizational (workplace), system (healthcare) level, or in combination have been developed and evaluated. A typology of these interventions is presented in Table 2. The nature of workplace involvement is often difficult to determine in these studies (being the place of delivery, the operator-facilitator and/or the target of the intervention). Even when interventions are indicated as workplace-based or oriented, most are in fact individually targeted [78–81].

**Table 2:** Intervention strategies from the scientific literature to improve outcomes for workers with mental health disorders

Level of intervention	Class of intervention	Examples of intervention
Worker level	Psychological intervention	Cognitive and/or behavioral therapy Skills training (stress inoculation training) Gradual exposure Problem solving therapy Occupational therapy Job coaching
	Physical intervention	Relaxation training Exercise
	Pharmacological intervention	Medication
Workplace level	Altering material conditions	Reduce physical exposures Reduce chemical exposures
	Altering work schedules	Working hours Working shifts Work intensity Work pace and deadlines Rest breaks
	Altering work organization	Reduce psychological job demands Reduce problematic social factors Assess efforts and provide rewards Adjust responsibilities Alter processes and procedures Alter team organization and structure
Healthcare provider level	Provide enhanced care	Strengthen work focus in primary care Strengthen work focus in psychiatric care Strengthen role of occupational physician
	Improve care coordination	Integrated case management across disciplines Coordination of care

For a listing of applicable reference citations, see Table 3

Several systematic reviews attempted to synthetize the evidence from original studies, but the heterogeneity of interventions and outcomes precludes any single conclusion (Table 3). For example, a Cochrane systematic review [80] identified 5 workplace-based interventions intended to promote return to work for workers will mental ill-health [82–86], which demonstrated a significant improvement in time until first RTW (HR 2.64, 95 % CI 1.41–4.95), but no impact on sustained RTW (HR 0.79, 95 % CI 0.54–1.17). All studies had been conducted in the Netherlands. Two main components of these interventions are worth mentioning. Firstly, a cognitive perspective of the RTW process was adopted, with the provision of a psycho-educational component including empowerment, problem resolution skills and self-help competencies. Secondly, a collaborative / participative approach was adopted, aiming at achieving a consensus between the supervisor and the worker about work accommodations and the RTW process. Both components required professionals (occupational therapist, RTW coordinator) who were trained about addressing workplace issues and the work organization, in addition to the principles of cognitive—behavioral therapy. The authors of a recent review concluded that there was at best low quality evidence on the effectiveness of workplace interventions for workers with mental health problems [80].

**Table 3:** Conclusions of systematic reviews of the scientific literature concerning mental health and work outcomes

References	Description	Primary conclusions
Furlan et al. [79]	Review of intervention practices for depression in the workplace	Evidence was graded as “very low” for all outcomes identified; therefore, no interventions were recommended
Nieuwenhuijsen et al. [80]	Review of interventions to improve return-to-work (RTW) after depression	Moderate quality evidence that adding a work-directed intervention to clinical intervention reduces number of days on sick leave; moderate quality evidence that enhancing primary or occupational care with cognitive behavioral therapy reduces days on sick leave
Arends et al. [94]	Review of interventions to facilitate RTW after adjustment disorders	No randomized controlled trials (RCTs) were found of employee assistance programs; Eight studies focused on the work environment; moderate-quality evidence that problem solving therapy significantly enhanced partial RTW at one-year follow-up
Montano et al. [95]	Review of effects of organizational-level interventions at work on employee health	Success rates were higher among more comprehensive interventions tackling material, organizational and work-time related conditions simultaneously
Bhui et al. [96]	Review of ways to manage stress at work (A summary of existing reviews reporting on anxiety, depression, and absenteeism)	Individual interventions had a greater effect size for individual-level outcomes; there was mixed evidence on the effectiveness of organizational interventions on absenteeism; there was clear evidence that employer-based physical activity promotion has a small effect on total absenteeism; Some interventions paradoxically led to deterioration in mental health and absenteeism
Kuoppala et al. [97]	Review of evidence for workplace health promotion on job well-being, work ability, and absenteeism	Sickness absence is reduced by activities promoting healthy lifestyle and ergonomics
Lamontagne et al. [98]	Review of the evidence supporting job stress interventions	Individual-focused approaches are effective at the individual level, but these interventions have no measurable impact at the organizational level
Odeen et al. [99]	Review of active workplace interventions to reduce sickness absence	One early intervention in employees with mild to severe depressive complaints and high risk of future long-term sickness absence proved to be effective in preventing/reducing both sickness absence and depressive complaints
Murta et al. [87]	Review of process evaluations in job stress management programs	Fewer than half of studies linked process evaluation to outcome evaluation; process relevant variables were recruitment, intervention dose received, participants’ attitudes toward intervention, and program reach

#### Limitations in Work-Related Mental Health Studies

Several limitations of the published workplace-related mental health studies must be emphasized. There is a lack of theoretical reasoning as the basis for the proposed interventions, especially in relation to addressing the work environment [78, 87]. The nature of the impairments and work limitations due to MH conditions were not well described. The concept of “early intervention” that is supported in most musculoskeletal condition—related RTW research has actually led to worse results in at least one mental health-related study, perhaps suggesting that work resumption should be delayed in some instances until significant MH improvement or stabilization has occurred [84]. As related to persistent mental health conditions there is a large discrepancy between psychosocial work factors identified as related to work disability in MH conditions, and the paucity of interventions at the organizational (workplace) level that might actually mitigate these factors.

In the few studies that reported process evaluations, implementation obstacles have included interventions being too complex, delivered too early in the course of the MH condition, or too time-consuming [84, 86]. The risks for the worker with MH problems of discussing psychosocial work factors with workplace actors are also rarely discussed, which is concerning, given the importance of workplace collaboration emphasized in many RTW interventions [83, 85, 88, 89].

#### Observations from the Grey Literature

This literature emphasizes that interventions often are delayed until it is too late to exert any lasting effect, key stakeholders are left out, and different institutions and services tend to work in isolation [73]. Therefore, the OECD advocates for a shift toward earlier (but not too early), more integrated and front-line interventions, in order to avoid work exclusion of people with mental ill health [73]. Line managers and general practitioners have been identified as best placed to identify work-related issues, to address impacts and implications, and to involve other professionals as necessary [73]. However, they need improved skills to work with employees experiencing mental health challenges, operational guidelines, and better tools to assist them with both identification and triage and referral processes. Anti-stigma policies can create a better environment to address employee concerns and challenges directly [73].

While a number of potential job accommodations are described in the grey literature related to mental health conditions, there are very few investigations of the effects of these work adjustments in the scientific literature. The roles of frontline managers and work organization are studied more often, but the results are also quite limited (Table 4).

**Table 4:** Sample recommendations (grey literature) for workplace approaches to reducing mental health absenteeism

Source	Recommendation
American College of Occupational and Environmental Medicine [100]	Use comprehensive approaches that span injury prevention, health promotion, and accommodation Provide primary prevention through mental health and resilience promotion Identify and modify sources of stress and other relevant risk factors, reduce stigma Provide employee education, voluntary screening, supervisor training, and employee assistance programs Facilitate early detection and treatment before sickness absence or job loss occurs Establish referral pathways to find evidence-based practitioners experienced in workplace issues Provide workplace accommodations for disability prevention and return to work Engage on-site medical personnel to actively support treatment adherence Ensure management commitment to an integrated workplace approach for dealing with depression Evaluate programs periodically for most effective coordination of mental health problems
American College of Occupational and Environmental Medicine [9]	Ensure that medical determinations of ability to work are based on accurate job information Recognize potential negative life impacts of a prolonged work absence Early disability prevention efforts are best Educate employees about the benefits of an early return to work Insure appropriate medical treatment of mental health conditions
Job Accommodation Network [101]	Give practical guidance on workplace accommodations to address specific functional problems at work Suggest potential accommodations for a range of mental conditions and provide case example illustrations
O’Day [102]	Use evidence-based Supported Employment (SE) for people with serious mental illnesses Provide a reasonably supportive workplace with flexibility and empathy
Waddell et al. [103]	Become involved in community efforts to provide accommodations for persons with mental health problems EAP programs may help to reduce mental-health related work disability Use disease and case management, disability management, and early contact and improved communication Provide routine occupational health consultations at the workplace for employees with mental health disorders
Organization for Economic Cooperation and Development [104]	Address mental health stigma Manager training and support to respond to workers’ mental health issues, including toolkits Human resource professionals who provide education and support to managers about the RTW process
Organisation for Economic Cooperation and Development [104]	Avoid high levels of job stress Provide early response to sick leave Adopt strategies to avoid workplace conflicts and reduce stigma Insist on being an active part of a workers’ rehabilitation plan to achieve a sustained return to work Ensure adequate manager support and positive reinforcement Modified duty or partial sick leave may be an effective strategy to prevent total work absence
World Health Organization [105]	Increase general employee awareness of mental health issues Support employees at risk Provide early access to treatment for employees with mental health problems Reintegrate employees with a mental health problem into the workplace Effective accommodation should include supervisor orientation, modified work times, and co-worker support Potentially useful accommodations are flexible working hours, education, using selected co-workers as mentors Protect confidentiality Change job content

### Recommendations for Future Studies

Based on our review, input from the Special Panel and participants of the Hopkinton conference, several key research priorities emerge. There are some questions that may appear to be unique to specific chronic conditions, but upon careful consideration, many of these issues are variations of the major research needs we identified. For example, the observed variability of work disability factors across age cohorts parallels the variability within a single diagnostic category. However, there is still not sufficient evidence to conclude that all key principles of workplace-centered work disability prevention apply equally across all conditions, as a few studies suggest otherwise [90].

1. *Identifying mechanisms in the RTW process* Research should identify the mechanisms of a successful RTW process in the workplace, for those with chronic illnesses, across the lifespan, and develop robust theoretical models that guide workplace interventions. There is already a start with research that has identified the characteristics of success in workers with chronic health conditions who maintain employment, as well as studies using logic modelling (such as intervention mapping), and other methods to identify the process and systems of successful RTW processes or interventions. But current investigations do not address the challenges of persons with specific chronic conditions or workers across the lifespan. Workplace stakeholders should be involved from the outset in defining the problem and developing solutions.

2. *Identifying those at greatest risk of disability* We need research on how to effectively and efficiently identify workers with chronic illness, who are at risk for WD, addressing workers across the whole lifecourse, applicable to multiple conditions. This should consider not only workplace and individual factors, but also the influence of socioeconomic inequality, gender issues, and other factors that are relevant. And a longitudinal view is important—not only work disability prevention, initial return to work, but also maintaining work, quality of work life, and career progression as important outcomes in both young and older workers.

3. *Identifying possibilities for work accommodation and support* We need research to identify the accommodations and other workplace supports that lead to optimal WDP outcomes, and whether and how the most effective interventions differ by condition. Rather than relying on standardized, diagnosis –based interventions, there is an overarching need to insure that more interventions are individually-directed, worker-centered and workplace-focused, rather than proscriptive and externally generated.

4. *Determining appropriate strategies* Studies should determine whether different strategies are needed for chronic health conditions that are less visible, episodic, variable in impact, or highly influenced by comorbidity. The focus should be on conditions where symptoms or work impact are less visible and apparent to others, health conditions (such as rheumatoid arthritis or bipolar disorder) or symptoms that are episodic and affect work, or conditions that have a variable and sometimes unpredictable impact on work ability. Comorbidity effects are just starting to be appreciated, yet there are not effective workplace strategies to address their impact on WD. Cancer has a potentially unique situation in that chemotherapy leads to side-effects that can last for a long time, are often under-recognized by workers and their employers,—especially if the appearance of these side-effects is delayed for several years after a ‘cure’ has been achieved. This could be a significant opportunity for condition-specific education of supervisors and employers.

5. *Greater focus on work retention and sustainability* For most chronic health conditions, there is a need for greater focus on work retention/sustainability and career development and progression. Most studies have focused on the individual alone, but more interventions are needed that address work conditions, integrating a proactive approach to vocational adjustment. For post-retirement workers, understanding their career status, financial status, and how this relates to WDP interventions may be particularly important.

6. *Incorporating workplace (organizational) solutions* Rather than focusing on the worker alone, workplace WDP studies should also address workplace climate, attitudes, responses and readiness for change in relation to WDP chronic health conditions, even though these interventions will be more costly and time-consuming at the outset. Studies should specifically target new ways to enhance supportive environments through co-worker and supervisor training and education of organizational leadership about the challenges of work participation with chronic illness. Emerging studies are addressing workplace policies and practices, making an economic case for broader programs that improve workplace health and safety [91]. Parallel evaluations should address employment of persons with chronic health conditions affecting their ability to work.

7. *Recognition of stigma as a potential obstacle to work participation* Research should address the importance of stigma as an obstacle to work participation. We should learn more about the factors that lead to both overt and covert (subtle) work discrimination, and develop primary and secondary prevention approaches for this problem in relation to chronic health conditions. Although this is primarily considered with mental health conditions, it is likely to interfere with WDP in other conditions as well, particularly with conditions that are relatively new to the workplace such as early onset dementia, autoimmune disease in young persons, chronic HIV infection.

8. *Considering the broader health care and work disability systems* For chronic health conditions, workplace interventions need to be articulated within the broader healthcare and disability systems. Increased emphasis should be placed on integrated and sustainable involvement among employer, worker and health care providers. Investigation in some contexts and the Special Panel suggest the potential value of developing and testing a stepped care approach to WD in the workplace—where an initial simple workplace intervention is followed by a sequence of more intensive interventions if the initial approach is not successful [42].

9. *Clarifying responsibilities* From a public policy point of view, there is a need for greater clarification of the responsibilities of all primary actors and that financial incentives are clearly aligned to achieve desired outcomes [73]. Research studies could be designed to incorporate multiple stakeholders in data collection of potential risk factors or in the development and testing of new intervention strategies.

#### Avenues for Future Studies

The overview of the scientific and the grey literature also provided an opportunity to identify important avenues for future studies. Early screening by frontline actors (managers, general practitioners, and occupational physicians) should be developed and linked with appropriate (stepped and/or integrated) care. Frontline managers in the workplace should receive training and support in this respect, both for screening and accommodating workers. The effectiveness of job accommodation / work (re)organization strategies proposed in the grey literature should be evaluated. Eventually, these efforts should be integrated with usual mental health care and medical case management services to offer a more seamless intervention for workers.

## Conclusions

There are general principles of effective workplace WDP strategies that are consistent across chronic health conditions, and less information suggesting that there may be condition-specific differences in the most effective strategies. Many chronic conditions are not readily visible, variable in the associated symptoms and impact on work ability, result in stigma upon disclosure, and can lead to discrimination in the workplace, based on a specific diagnosis. At present, these factors limit the ability of employers to effectively accommodate, support, and maintain employment of affected workers. Studies of workers with chronic conditions who are able to maintain employment suggest that work organizational response, self-determined accommodations, work flexibility, and consistent supervisor and manager support are key to their success.

After reviewing the existing scientific literature on WDP, chronic health conditions, and work disability across the working life course, we have found remarkably little research to guide WDP practice. We did not address young workers with health conditions in depth, other chronic conditions (e.g., cardiovascular, infectious, metabolic, neurologic, and cognitive conditions), as we wanted to provide a few examples that would illustrate both differences and similarities. Although there may be important unique features for a particular condition that relate to WDP interventions (e.g., types of symptoms and specific functional limitations), our overall conclusion is that the basic principles of effective workplace-based WDP are most important.

Although the scientific and grey literatures tend to focus on personal determinants and diagnoses, and easily-implemented accommodations, there is ample evidence for the importance of organizational factors across many of these special situations. More broad recognition of the importance of work organizational responses, as opposed to greater clinical focus, is needed to better address WD prevention. Work organizational factors are occasionally emphasized in the grey literature, but are poorly represented in research; given their importance in solving WD problems, this is a priority for these special populations. For example, in what ways do specific organizational issues present a unique challenge for workers with a particular condition (such as cancer), and how can this be overcome? And, how do new work organizational structures, such as temporary, lone work and virtual work present unique challenges or opportunities for WDP for specific conditions or stages of the working life-course? Studies on supervisor response, informal and effective accommodations, and how best to address variable conditions and resulting fluctuations in work ability appear to be a priority. For the small employer, a challenge is finding and providing problem-solving resources that can be appropriately specific to a particular condition and work arrangement, for a relatively rare occurrence (work disability).

As more workers with chronic illness seek sustained employment, there is the potential for employers to face more demands for accommodations, costs related to maintaining employment of these workers, more challenges in figuring out who can work and when, and perhaps less predictability about who can stay employed in the long –term. These trends will lead to broader changes as some organizations will find ways to accommodate a broader range of different challenges. Employers who are able to meet these challenges will have access to a greater range of workers and talent, and may reap benefits in terms of affinity and loyalty.
